# Usefulness of Three-Dimensional Iodine Mapping Quantified by Dual-Energy CT for Differentiating Thymic Epithelial Tumors

**DOI:** 10.3390/jcm12175610

**Published:** 2023-08-28

**Authors:** Shuhei Doi, Masahiro Yanagawa, Takahiro Matsui, Akinori Hata, Noriko Kikuchi, Yuriko Yoshida, Kazuki Yamagata, Keisuke Ninomiya, Shoji Kido, Noriyuki Tomiyama

**Affiliations:** 1 Department of Radiology, Osaka University Graduate School of Medicine, 2-2 Yamadaoka, Suita-City 565-0871, Osaka, Japan; 2 Department of Pathology, Osaka University Graduate School of Medicine, 2-2 Yamadaoka, Suita-City 565-0871, Osaka, Japan; 3 Department of Artificial Intelligence Diagnostic Radiology, Osaka University Graduate School of Medicine, 2-2 Yamadaoka, Suita-City 565-0871, Osaka, Japan

**Keywords:** thymoma, thymic carcinoma, dual-energy CT, extracellular volume fraction, iodine effect

## Abstract

*Background*: Dual-energy CT has been reported to be useful for differentiating thymic epithelial tumors. The purpose is to evaluate thymic epithelial tumors by using three-dimensional (3D) iodine density histogram texture analysis on dual-energy CT and to investigate the association of extracellular volume fraction (ECV) with the fibrosis of thymic carcinoma. *Methods*: 42 patients with low-risk thymoma (*n* = 20), high-risk thymoma (*n* = 16), and thymic carcinoma (*n* = 6) were scanned by dual-energy CT. 3D iodine density histogram texture analysis was performed for each nodule on iodine density mapping: Seven texture features (max, min, median, average, standard deviation [SD], skewness, and kurtosis) were obtained. The iodine effect (average on DECT180s—average on unenhanced DECT) and ECV on DECT180s were measured. Tissue fibrosis was subjectively rated by one pathologist on a three-point grade. These quantitative data obtained by examining associations with thymic carcinoma and high-risk thymoma were analyzed with univariate and multivariate logistic regression models (LRMs). The area under the curve (AUC) was calculated by the receiver operating characteristic curves. *p* values < 0.05 were significant. *Results*: The multivariate LRM showed that ECV > 21.47% in DECT180s could predict thymic carcinoma (odds ratio [OR], 11.4; 95% confidence interval [CI], 1.18–109; *p* = 0.035). Diagnostic performance was as follows: Sensitivity, 83.3%; specificity, 69.4%; AUC, 0.76. In high-risk thymoma vs. low-risk thymoma, the multivariate LRM showed that the iodine effect ≤1.31 mg/cc could predict high-risk thymoma (OR, 7; 95% CI, 1.02–39.1; *p* = 0.027). Diagnostic performance was as follows: Sensitivity, 87.5%; specificity, 50%; AUC, 0.69. Tissue fibrosis significantly correlated with thymic carcinoma (*p* = 0.026). *Conclusions*: ECV on DECT180s related to fibrosis may predict thymic carcinoma from thymic epithelial tumors, and the iodine effect on DECT180s may predict high-risk thymoma from thymoma.

## 1. Introduction

Thymic epithelial tumors account for 0.05% of all malignant tumors and 47% of anterior mediastinal tumors. They occur equally in men and women and occur in a wide age range (especially in the 40–60 age group). Thymic carcinoma accounts for 10–20% of thymic epithelial tumors [1,2]. Thymic epithelial tumors are classified according to the WHO classification into type A, type AB, type B1, type B2, type B3 thymomas, and thymic carcinoma. Types A, AB, and B1 are classified as low-risk thymomas, while types B2 and B3 are classified as high-risk thymomas [3]. Low-risk thymomas are more likely to be completely resected without the need for adjuvant chemotherapy. However, multidisciplinary treatment with a combination of surgical resection, radiation therapy, and chemotherapy before and after surgery is required for patients with high-risk thymoma and/or thymic carcinomas [2,4].

Computed tomography (CT) and/or magnetic resonance (MR) images are crucial for the pretreatment evaluation of thymic epithelial tumors. On CT and MRI, thymic carcinomas are more likely to present with irregular margins, necrosis or cystic degeneration, heterogeneous contrast enhancement, lymphadenopathy, and macrovascular invasion [5]. However, subjective evaluation has problems such as difficulty in making an accurate diagnosis due to many overlapping image findings and poor reproducibility among readers.

Various quantitative evaluations are being attempted to make a more appropriate and reproducible diagnosis, such as CT perfusion imaging, which has been reported to be useful in distinguishing thymoma from other anterior mediastinal malignancies [6,7]. MRI has reported that the ADC map and its histogram analysis may be useful in differentiating thymic carcinomas [6,8,9]. CT and MRI radiomics analysis may be useful in predicting the pathologic classification and staging of thymic epithelial tumors [2,4,10]. Dual-energy CT might be one of the non-invasive CT techniques for quantitative analyses of thymic epithelial tumors. Dual-energy CT shows significant differences in iodine-related Hounsfield units (IHUs) and iodine concentrations (ICs) between low-risk thymoma, high-risk thymoma, and thymic carcinoma [6,11]. However, as far as we could find, there are no reports of 3-dimensional (3D) iodine density histogram texture analysis on dual-energy CT.

The extracellular volume fraction (ECV) represents the ratio of extracellular fluid volume to empty volume and is considered an indicator reflecting tissue fibrosis and edema, and studies have made correlations with fibrosis in the myocardium, liver, and pancreas, but no studies have evaluated fibrosis in thymic epithelial tumors [12,13,14].

We hypothesized that a 3D histogram texture analysis of dual-energy CT would provide a more accurate diagnosis of thymic epithelial tumors, and the evaluation of ECV would provide clues for finding associations of fibrosis with thymic carcinoma. This study evaluated whether 3D quantitative values from dual-energy CT can distinguish thymic carcinoma and/or high-risk thymoma from low-risk thymoma and investigated the association of ECV with fibrosis of thymic carcinoma.

## 2. Materials and Methods

This study was approved by the ethical review committee of Osaka University (No. 22181) and was conducted in accordance with the principles of the Declaration of Helsinki. Informed consent was not obtained as this was a retrospective review of images and records.

### 2.1. Patients

**Figure 1:**
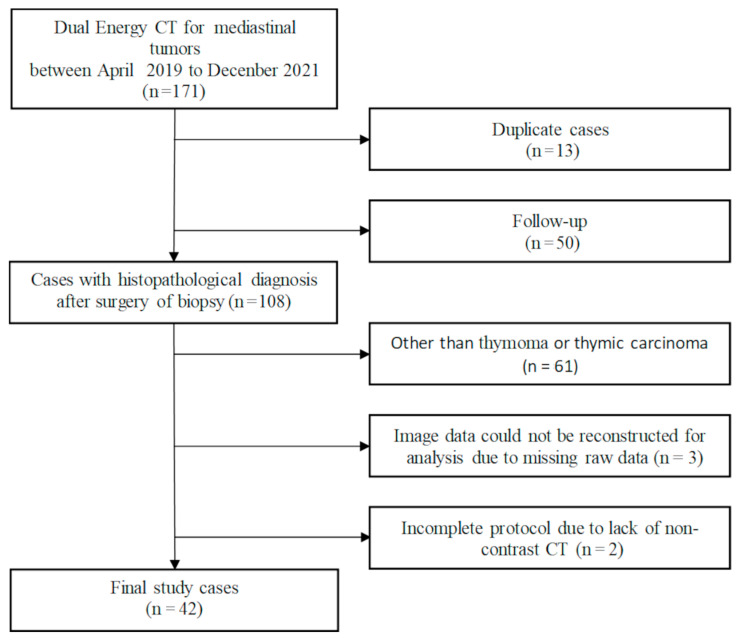
Flowchart of patient selection.

The inclusion criteria were as follows: Surgery (*n* = 40) or biopsy (*n* = 2) performed between April 2019 and December 2021 and histological diagnosis was confirmed, and dual-energy non-contrast and dynamic contrast CT thin slice data must be available. Of the 171 patients, 13 were excluded because of duplicate cases and 50 because only follow-up was available, while the remaining 108 with histopathological diagnoses were included. Of the 108 patients with the diagnosis, 61 patients were excluded because the pathological diagnosis was not thymoma or thymic carcinoma, 3 because there was missing raw data needed for reconstruction for analysis, and 2 because protocols were incomplete due to the lack of non-contrast CT. Finally, 42 patients with low-risk thymoma (*n* = 20), high-risk thymoma (*n* = 16), and thymic carcinoma (*n* = 6) were included in our study (Figure 1).

### 2.2. Dual-Energy CT protocols

Thymic epithelial tumors were examined by dual-energy dynamic multiphase CT using a 64-ch Discovery CT750 HD (GE Healthcare, Milwaukee, WI, USA). The following CT protocol was used: 0.625 mm of detector collimation; 1.375 of detector pitch; 0.5 s of gantry rotation period; 512 × 512 pixels of matrix size; fast kV switching mode (80 kVp and 140 kVp) of X-ray voltage; auto mA of tube current; 34.5-cm field of view; 1.25-mm thickness; a standard kernel; and 30% adaptive statistical iterative reconstruction. Unenhanced CT and enhanced CT with 180 s-delayed scans using IOHEXOL (iodine contrast material of 300 mg/cc; GE Healthcare Pharma, Tokyo, Japan) were performed. The dose of contrast material per patient was decided on the basis of 2 mL per weight. The mean contrast volume was 122.3 mL ± 17.1 (range, 97–144 mL). Radiation doses were as follows: CT dose index volumes (CTDIvol) of unenhanced and enhanced CT were 11.72 ± 3.2 and 10.3 ± 2.4 mGy; dose-length product (DLP) of unenhanced and enhanced CT was 551.0 ± 154.4 and 296.9 ± 77.0 mGy-cm, respectively.

### 2.3. Image Analysis

Gemstone Spectral Imaging (GSI) image datasets from dual-energy CT were transferred to Advantaged Workstation VolumeShare7 (GE Healthcare). After the reference standard for tumor boundaries was determined by agreement between two thoracic radiologists (M.Y. and S.D. with 23 and 8 years of experience, respectively) using multi-planar reconstruction images of CT, the 3D volume of interest (VOI) of the tumor was semiautomatically extracted using a GSI VolumeViewer, and manual corrections were made in cases where the 3D VOI and reference standard differed. Then, the final 3D VOI was applied to an iodine density image. Seven texture features (maximum, minimum, median, average, standard deviation [SD], skewness, and kurtosis) were calculated using an iodine density histogram derived from each voxel datum. The iodine effect was calculated as follows: Iodine concentration in enhanced dual-energy CT − iodine concentration in non-enhanced dual-energy CT. VOI on 3D iodine density mapping was set in each nodule as large as possible to reduce the effect of tumor heterogeneity. Assuming that the extracellular fluid iodine contrast agent is evenly distributed in the extracellular fluid space in the equilibrium phase, the ECV is obtained by correcting the ratio obtained from the tissue iodine value and the aortic iodine value, which are differenced in the equilibrium and non-enhanced phases on CT, by the hematocrit value. ECV was calculated using the following formula: ECV = (1 − hematocrit) × (iodine density in each nodule/iodine density in each aorta) (%). The extracellular volume fraction can measure the extracellular space as a percentage of non-cellular tissue volume.

### 2.4. Histological Evaluation

Paraffin-embedded specimens were cut into 4-μm sections and stained with hematoxylin and eosin for histological diagnosis and with Masson’s trichrome for evaluation of fibrosis using standard protocols by pathologists. Histological diagnosis was classified into six subtypes (thymoma type A, AB, B1, B2, B3, and thymic carcinoma) based on the 2015 WHO classification [3]. The evaluation of fibrosis was subjectively rated by one pathologist (T.M. with 19 years of experience) on a three-point scale (1 = weak, 2 = medium, 3 = strong). Since no criteria exist for assessing fibrosis in thymic epithelial tumors, the following fibrosis grades in the fibrosis focus of breast cancer [15] were used: Grade 1, a large number of fibroblasts with a small number of collagen fibers; Grade 3, mainly composed of collagen fibers, mostly hyalinized; and Grade 2, intermediate between Grade 1 and 3, with fibroblasts and collagen fibers intermingled in various ratios.

### 2.5. Statistical Analysis

MedCalc (Version 13.1.2.0–64 bit, Frank Schoonjans, Mariakerke, Belgium) was used for statistical analyses. Quantitative values among the three groups (low-risk thymoma, high-risk thymoma, and thymic carcinoma) based on the WHO classification were compared using Friedman’s test. Subjective evaluations of pathological fibrosis within the tumor were compared among the three groups using the Mann–Whitney U test. Associations between thymic carcinoma and nine parameters (maximum, minimum, median, mean, SD, skewness, kurtosis, iodine effect, and ECV) were evaluated using univariate and multivariate logistic regression analyses. For each feature, the cutoff value that yielded the largest difference in the number of patients with and without thymic carcinoma was determined using the receiver-operating characteristic (ROC) method. Optimal thresholds were determined for each variable separately using the Youden index (the highest sum of sensitivity and specificity). Associations between thymic carcinoma and each binary group designated by the cutoff value for the nine parameters were evaluated by univariate logistic regression analysis. Significant parameters identified by univariate analysis were included in multiple logistic regression (stepwise method; a *p*-value of 0.05 or less was used for entry into the model and a *p*-value greater than 0.1 was selected for removal). The diagnostic performance was evaluated by sensitivity, specificity, and the area under the receiver operator characteristic curve (AUC). *p* < 0.05 was considered significant.

## 3. Results

### 3.1. Study Population

Patient characteristics were as follows: 42 patients (20 male and 22 female) with a thymic epithelial tumor had a mean age of 58.3 (range, 21–83) years old: 20 low-risk thymoma (type A; *n* = 5, AB; *n* = 9, B1; *n* = 6), 16 high-risk thymoma (type B2; *n* = 12, B3; *n* = 4), and 6 thymic carcinoma. Two patients with thymic carcinoma underwent biopsy only, and the remaining forty patients underwent surgery.

### 3.2. Quantitative Date

**Figure 2:**
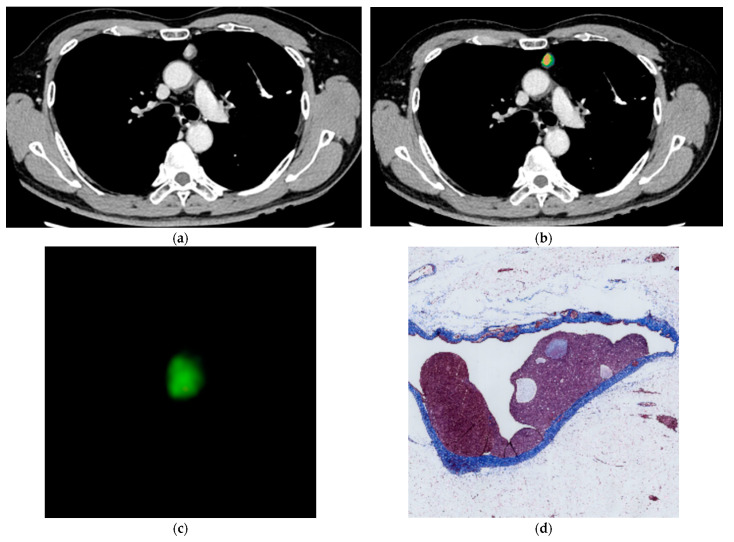
72 y.o. male with low-risk thymoma (thymoma AB). The quantitative parameters are as follows: max: 4.0 mg/cc, min: −2.9 mg/cc, median: 1.4 mg/cc, average: 1.44 mg/cc, SD: 1.498 mg/cc, skewness: −0.32, kurtosis: 2.52, iodine effect: 1.56 mg/cc, and ECV: 18.79%. (**a**) Axial contrast-enhanced CT image shows an anterior mediastinal tumor 15 mm in diameter. (**b**) Color map of dual-energy CT. (**c**) 3D reconstruction with volume rendering. (**d**) Histopathological image of Masson’s trichrome staining. Subjective pathological score of fibrosis was grade 1 within tumor.

**Figure 3:**
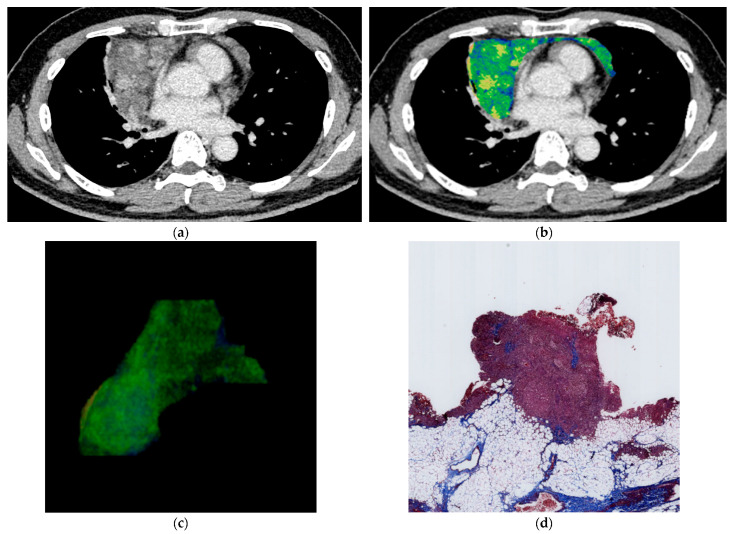
46 y.o. male with high-risk thymoma (thymoma B2). The quantitative parameters are as follows: max: 5.7 mg/cc, min: −2.8 mg/cc, median: 7.0 mg/cc, average: 0.76 mg/cc, SD: 0.75 mg/cc, skewness: 0.87, kurtosis: 4.13, iodine effect: 0.84 mg/cc, ECV: 9.48%. (**a**) Axial contrast-enhanced CT image shows an anterior mediastinal tumor 135 mm in diameter. (**b**) Color map of dual-energy CT. (**c**) 3D reconstruction with volume rendering. (**d**) Histopathological image of Masson’s trichrome staining. Subjective pathological score of fibrosis was grade 1 within tumor.

**Figure 4:**
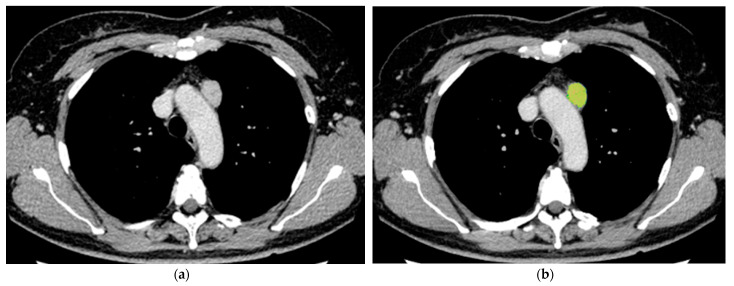

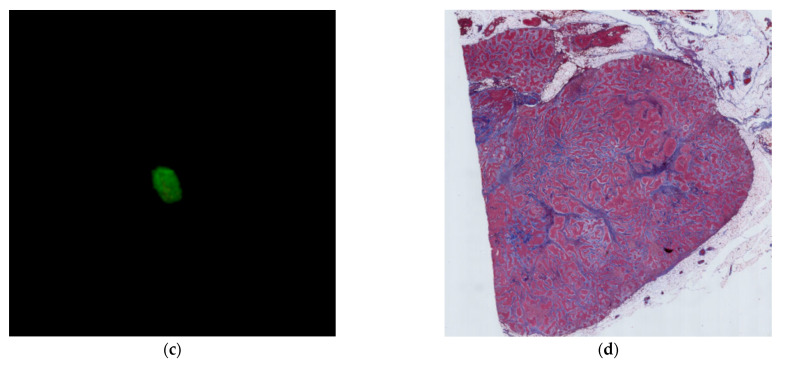
56 y.o. female with thymic carcinoma. The quantitative parameters are as follows: max: 3.3 mg/cc, min: −2.9 mg/cc, median: 2.3 mg/cc, average: 1.84 mg/cc, SD: 1.18 mg/cc, skewness: −2.0, kurtosis: 6.43, iodine effect: 1.83 mg/cc, and ECV: 27.87%. (**a**) Axial contrast-enhanced CT image shows an anterior mediastinal tumor 25 mm in diameter. (**b**) Color map of dual-energy CT. (**c**) 3D reconstruction with volume rendering. (**d**) Histopathological image of Masson’s trichrome staining. Subjective pathological score of fibrosis was grade 2 within tumor.

Table 1 shows the mean ± SD of the nine quantitative features (maximum, minimum, median, average, SD, skewness, kurtosis, iodine effect, and ECV) for each pathological diagnosis (Figure 2, Figure 3 and Figure 4). Significant differences were found in ECV between the groups of high-risk thymoma and thymic carcinoma, and high-risk thymoma and low-risk thymoma, respectively. The cutoff values of quantitative features for diagnostic prediction and the mean ± SD of values for each binary group obtained by ROC analysis are shown in Table 2. For the comparison between thymic carcinoma and thymoma, score = 1 for thymic carcinoma and score = 0 for thymoma. For high-risk thymoma vs. low-risk thymoma, score = 1 for high-risk thymoma and score = 0 for low-risk thymoma.

### 3.3. Predictive Performance for Thymic Carcinoma Using Quantitative Features

In examining the quantitative features to differentiate thymic carcinoma from thymoma, the univariate logistic analysis showed that SD > 0.84 mg/cc and ECV > 21.47% on contrast CT were useful in predicting thymic carcinoma from thymic epithelial tumors (odds ratio [OR], 10 and 11.4; 95% confidence interval [CI], 1.05–95.5 and 1.18–109; *p* = 0.046 and 0.035, respectively). Multivariate logistic analysis showed that ECV > 21.47% was useful in predicting thymic carcinoma from thymoma. (OR, 11.4; 95% CI, 1.18–109; *p* = 0.035) (Table 3). Diagnostic performance was as follows: Sensitivity, 83.3% (five of six patients); specificity, 69.4% (25 of 36 patients); AUC, 0.76 (95% CI, 1.18–109).

In examining the quantitative features to differentiate high-risk thymoma from thymoma, univariate logistic analysis showed that an average of ≤1.61 mg/cc, iodine effect of ≤1.31 mg/cc, and ECV of ≤ 21.47% were useful in predicting high-risk thymoma from thymoma (OR, 10.7 and 5.7; 95% CI, 1.09–91.4,1.25–39.1, and 1.02–32.1; *p* = 0.041, 0.027, and 0.047, respectively). Multivariate logistic analysis showed that an iodine effect of ≤1.31 mg/cc was predictive of thymoma to high-risk thymoma (OR, 7; 95% CI, 1.02–39.1; *p* = 0.027) (Table 4). Diagnostic performance was as follows: Sensitivity, 87.5% (14 of 16 patients); specificity, 50% (10 of 20 patients); AUC, 0.69 (95% CI, 1.02–39.1).

### 3.4. Pathological Evaluation of Fibrosis within the Tumor

A subjective assessment of pathological fibrosis within the tumor according to WHO classification was performed. Fibrosis within the tumor was evaluated pathologically in thymic carcinoma (four cases) and thymoma (36 cases) excluding two cases that were not operated on and only biopsied. Pathological subjective scores [mean ± SD (range)] were as follows: Low-risk thymoma [1.45 ± 0.62 (1–3)], high-risk thymoma [1.56 ± 0.63 (1–3)], and thymic carcinoma [2.25 ± 0.50 (2–3)]. Subjective scores by the pathologist were significantly higher in cases with thymic carcinoma than in those with low-risk and high-risk thymoma (*p* = 0.026) (Figure 2, Figure 3 and Figure 4).

## 4. Discussion

We have demonstrated that 3D histogram texture analysis in dual-energy CT can predict thymic carcinoma by ECV and high-risk thymoma by the iodine effect, respectively. 3D histogram texture analysis has the potential to be a useful non-invasive diagnostic tool for differentiating thymic epithelial tumors, especially in cases where surgery or biopsy is not feasible. The prediction of thymic epithelial tumors is useful for deciding the management, including postoperative radiotherapy and chemotherapy in a clinical setting, because patients with high-risk thymoma or thymic carcinoma have lower 5- and 10-year overall survival rates than those with low-risk thymoma [16,17,18].

ECV using dual-energy CT-enabled prediction of thymic carcinoma in the present study. ECV is defined by measurements of the extracellular matrix and intracapillary plasma volume and is said to reflect tissue fibrosis and edema [19]. In the present study, subjective fibrosis assessment scores by the pathologist were significantly higher in thymic carcinoma than in thymomas. Generally, cancer-associated fibroblasts (CAFs) are abundant in malignant tumors and are an important factor in promoting malignant tumor growth and invasion. A central role in fibrosis of the cancer stroma is associated with CAF proliferation [20]. On the other hand, Chang et al. reported that ECV measured by T1 mapping of MRI of thymic epithelial tumors was significantly higher in thymic carcinomas than in thymomas, and in thymic epithelial tumors with fewer lymphocytes (thymoma types A, B3, and thymic carcinoma) than in thymic epithelial tumors with abundant lymphocytes (thymomas types AB, B1, and B2) [21]. Lymphocyte abundance is not neatly divided between high- and low-risk thymomas, which may explain why there was no significant difference in ECV between high- and low-risk thymomas in our study. Moreover, both fibrosis due to the presence of CAFs and fewer lymphocytes in the tumors may result in higher ECV in thymic carcinoma.

Yan et al. reported that several 2D parameters of dual-energy CT: Mean iodine concentration (mg/mL), mixed Hounsfield unit (MHU; CT attenuation value in postcontrast enhanced HU), and mean iodine-related HU (MH −virtual non-contrast HU) were significantly higher in low-risk thymomas than in high-risk thymomas and thymic carcinomas [22]. The present study was different from previous studies in that 3D analysis was used instead of 2D analysis, and in addition, the iodine effect was used rather than the iodine concentration itself. The iodine concentration on non-enhanced CT was ideally regarded as almost 0 μg/cm^3^ because there was no measurable iodine. A monochromatic energy projection can be synthesized by the weighted sum of material density projections using their corresponding mass attenuation coefficients at a given energy as the weighting factors, resulting in providing the estimated material densities [23,24]. The actual iodine value in non-enhanced CT is not always zero due to the point where the iodine–water material decomposition is based on the binarization concept of iodine or water, and mainly due to the effect of image noise. However, the iodine effect, which subtracts the pre-contrast iodine concentration from the postcontrast iodine concentration, is more accurate in that the effect of background image factors such as noise can be excluded. Moreover, considering the heterogeneity of tumors, 3D analysis of the entire tumor is considered superior to 2D analysis using manual regions of interest in terms of reproducibility and objectivity. High-risk thymomas have a higher frequency of necrosis, cystic degeneration, and heterogeneous contrast enhancement than low-risk thymomas [5,25,26]. The significantly lower iodine effect in high-risk thymomas compared to low-risk thymomas may reflect cystic degeneration, necrosis, or hemorrhage of the tumor, which may have resulted in weak iodine accumulation.

Recently, photon-counting CT has been clinically used as a promising new technology. Photon-counting CT can count the number of incident photons and measure photon energy. This allows for an improved contrast-to-noise ratio, improved spatial resolution, optimized spectral imaging, reduced radiation exposure, image reconstruction with a higher resolution, the correction of beam-hardening artifacts, the optimization of contrast agent use, and multi-energy analysis, as well as dual-energy CT [27]. Compared to dual-energy CT, PCT-CT is expected to provide more accurate quantitative data because of the high spatial resolution without electronic noise and with improved tissue contrasts. As future research topics, we are considering the possibility of using image analysis with multi-energy analysis using photon counting CT to predict prognosis and pathology with higher accuracy and predict the relationship between the molecular and metabolic levels of thymic epithelial tumors. Moreover, CT images provide essentially unique diagnostic information and play a useful role in developing a treatment strategy. However, it is often difficult to differentiate thymic epithelial tumors from lymphomas due to overlaps of image findings. If PCT-CT can accurately distinguish between them, it will be of high clinical utility because we can rule out lymphomas before surgery. It would be interesting to conduct research using quantitative analysis by PCT-CT for the differentiation of thymic epithelial tumors including lymphoma.

Our study has several limitations. First, the small number of patients in this study may have influenced on results. In particular, the number of thymic carcinomas was too small because of its scarcity, and the study was conducted at a single institution. External validation using a larger cohort is needed to acquire high-level evidence in a clinical setting. Second, two of the six thymic carcinoma cases were excluded from the pathological subjective evaluation because of only having a biopsy. The 3D images were analyzed, but only a visual assessment of pathological fibrosis was performed. Although 3D evaluation of fibrosis in pathological specimens would be ideal, it is clinically very difficult because making an accurate comparison of CT images with pathological specimens is difficult due to postoperative specimen shrinkage and evaluation of the entire surface of a pathological specimen is impossible.

In conclusion, in 3D histogram texture analysis of dual-energy CT, ECV could predict thymic carcinoma from thymic epithelial tumors, and the iodine effect could predict high-risk thymoma from low-risk thymoma. Quantitative analysis using dual-energy CT is a non-invasive diagnostic tool to differentiate thymic epithelial tumors, which may be helpful for determining patients’ management.

## Figures and Tables

**Table 1:** Quantitative values according to WHO classification.

	Mean ± SD		
	Low-Risk Thymoma	High-Risk Thymoma	Thymic Carcinoma
Maximum	5.61 ± 3.64	6.02 ± 5.05	4.67 ± 2.93
Minimum	−2.87 ± 0.047	−2.89 ± 0.034	−2.9 ± 0
Median	1.59 ± 0.67	1.54 ± 0.47	1.9 ± 0.35
Average	1.46 ± 0.55	1.35 ± 0.44	1.65 ± 0.29
SD	0.80 ± 0.28	0.86 ± 0.23	0.97 ± 0.18
Skewness	−0.97 ± 1.36	−1.01 ± 1.68	−1.61 ± 0.78
Kurtosis	8.28 ± 3.87	9.69 ± 8.09	6.69 ± 3.56
Iodine effect	1.31 ± 0.51	1.20 ± 0.36	1.61 ± 0.26
ECV	20.49 ± 7.22	18.65 ± 5.97	25.58 ± 4.67

SD: Standard deviation, ECV: Extracellular volume.

**Table 2:** Cutoff values of quantitative features for predicting diagnosis and values of binarized groups.

	Thymic Carcinoma vs. Thymoma			High-Risk Thymoma vs. Low-Risk Thymoma		
Features	N	Cut-Off Value	Mean ± SD	N	Cut-Off Value	Mean ± SD
Maximum						
Score = 0	24		7.56 ± 4.54	18		8.42 ± 4.75
Score = 1	18	≤3.6	3.06 ± 0.41	18	≤3.9	3.16 ± 0.51
Minimum						
Score = 0	8		−2.8	8		−2.8
Score = 1	34	≤−2.9	−2.9	28	≤−2.9	−2.9
Median						
Score = 0	22		1.21 ± 0.40	10		2.26 ± 0.31
Score = 1	20	>1.6	2.06 ± 0.35	26	≤1.7	1.30 ± 0.41
Average						
Score = 0	29		1.20 ± 0.34	9		2.06 ± 0.29
Score = 1	13	>1.61	1.98 ± 0.27	27	≤1.61	1.20 ± 0.35
SD						
Score = 0	25		6.83 ± 1.13	15		6.19 ± 0.99
Score = 1	17	>8.41	10.83 ± 1.97	21	>7.41	9.72 ± 2.31
Skewness						
Score = 0	31		−0.65 ± 1.41	30		−0.71 ± 1.47
Score = 1	11	≤−2	−2.29 ± 0.29	6	≤−2.14	−2.39 ± 0.29
Kurtosis						
Score = 0	31		10.41 ± 5.65	7		2.91 ± 0.82
Score = 1	11	≤4.78	3.33 ± 0.96	29	>4.13	10.30 ± 5.90
Iodine effect						
Score = 0	18		0.93 ± 0.26	12		1.76 ± 0.28
Score = 1	24	>1.2	1.60 ± 0.31	24	≤1.31	1.01 ± 0.27
ECV						
Score = 0	26		16.51 ± 4.87	11		27.05 ± 3.25
Score = 1	16	>21.47	27.04 ± 3.18	25	≤21.47	16.43 ± 4.95

SD: Standard deviation, ECV: Extracellular volume.

**Table 3:** Relationship of quantitative features with prediction of thymic carcinoma.

	Univariate Analysis					Multivariate Analysis				
Features	Thymoma (*n*)	Thymic Carcinoma (*n*)	Odds Ratio	95% Confidence Interval	*p* Value	Thymoma (*n*)	Thymic Carcinoma (*n*)	Odds Ratio	95% Confidence Interval	*p* Value
Maximum			3.1	0.5–19.5	0.219					
Score = 0 (*n* = 24)	22	2								
Score = 1 (*n* = 18)	14	4								
Minimum			1.68 × 10^8^		0.997					
Score = 0 (*n* = 8)	8	0								
Score = 1 (*n* = 34)	28	6								
Median			7	0.7–66.2	0.090					
Score = 0 (*n* = 22)	21	1								
Score = 1 (*n* = 20)	15	5								
Average			6	0.9–38.4	0.059					
Score = 0 (*n* = 29)	27	2								
Score = 1 (*n* = 13)	9	4								
SD			10	1.0–95.5	0.046					
Score = 0 (*n* = 25)	24	1								
Score = 1 (*n* = 17)	12	5								
Skewness			3.5	0.6–20.8	0.168					
Score = 0 (*n* = 31)	28	3								
Score = 1 (*n* = 11)	8	3								
Kurtosis			3.5	0.6–20.8	0.168					
Score = 0 (*n* = 31)	28	3								
Score = 1 (*n* = 11)	8	3								
Iodine effect			7.09 × 10^8^		0.998					
Score = 0 (*n* = 18)	18	0								
Score = 1 (*n* = 24)	18	6								
ECV			11.4	1.2–109.2	0.035			11.4	1.2–109.2	0.035
Score = 0 (*n* = 26)	25	1				25	1			
Score = 1 (*n* = 16)	11	5				11	5			

SD: Standard deviation, ECV: Extracellular volume.

**Table 4:** Relationship of quantitative features with prediction of high-risk thymoma.

	Univariate Analysis					Multivariate Analysis				
Features	Low-Risk Thymoma (*n*)	High-Risk Thymoma (*n*)	Odds Ratio	95% Confidence Interval	*p* Value	Low-Risk Thymoma (*n*)	High-Risk Thymoma (*n*)	Odds Ratio	95% Confidence Interval	*p* Value
Maximum			2.5	0.6–9.7	0.184					
Score = 0 (*n* = 18)	12	6								
Score = 1 (*n* = 18)	8	10								
Minimum			3	0.5–17.5	0.222					
Score = 0 (*n* = 8)	6	2								
Score = 1 (*n* = 28)	14	14								
Median			4.7	0.8–26.3	0.081					
Score = 0 (n = 10)	8	2								
Score = 1 (*n* = 26)	12	14								
Average			10	1.1–91.4	0.041					
Score = 0 (*n* = 9)	8	1								
Score = 1 (*n* = 27)	12	15								
SD			3.7	0.9–15.4	0.076					
Score = 0 (*n* = 15)	11	4								
Score = 1 (*n* = 21)	9	12								
Skewness			8.6	0.9–83.8	0.063					
Score = 0 (*n* = 30)	19	11								
Score = 1 (*n* = 6)	1	5								
Kurtosis			0.2	0.04–1.5	0.126					
Score = 0 (*n* = 7)	18	11								
Score = 1 (*n* = 29)	2	5								
Iodine effect			7	1.3–39.1	0.027			7	1.3–39.1	0.027
Score = 0 (*n* = 12)	10	2				10	2			
Score = 1 (*n* = 24)	10	14				10	14			
ECV			5.7	1.0–32.1	0.047					
Score = 0 (*n* = 11)	9	2								
Score = 1 (*n* = 25)	11	14								

SD: Standard deviation, ECV: Extracellular volume.

## Data Availability

The data presented in this study are available upon request from the corresponding author. The data are not publicly available due to restrictions on privacy.
